# The implementation leadership scale (ILS): development of a brief measure of unit level implementation leadership

**DOI:** 10.1186/1748-5908-9-45

**Published:** 2014-04-14

**Authors:** Gregory A Aarons, Mark G Ehrhart, Lauren R Farahnak

**Affiliations:** 1 Department of Psychiatry, University of California, San Diego, La Jolla, CA, USA; 2 Child and Adolescent Services Research Center, San Diego, CA, USA; 3 Department of Psychology, San Diego State University, San Diego, CA, USA

## Abstract

**Background:**

In healthcare and allied healthcare settings, leadership that supports effective implementation of evidenced-based practices (EBPs) is a critical concern. However, there are no empirically validated measures to assess implementation leadership. This paper describes the development, factor structure, and initial reliability and convergent and discriminant validity of a very brief measure of implementation leadership: the Implementation Leadership Scale (ILS).

**Methods:**

Participants were 459 mental health clinicians working in 93 different outpatient mental health programs in Southern California, USA. Initial item development was supported as part of a two United States National Institutes of Health (NIH) studies focused on developing implementation leadership training and implementation measure development. Clinician work group/team-level data were randomly assigned to be utilized for an exploratory factor analysis (n = 229; k = 46 teams) or for a confirmatory factor analysis (n = 230; k = 47 teams). The confirmatory factor analysis controlled for the multilevel, nested data structure. Reliability and validity analyses were then conducted with the full sample.

**Results:**

The exploratory factor analysis resulted in a 12-item scale with four subscales representing proactive leadership, knowledgeable leadership, supportive leadership, and perseverant leadership. Confirmatory factor analysis supported an *a priori* higher order factor structure with subscales contributing to a single higher order implementation leadership factor. The scale demonstrated excellent internal consistency reliability as well as convergent and discriminant validity.

**Conclusions:**

The ILS is a brief and efficient measure of unit level leadership for EBP implementation. The availability of the ILS will allow researchers to assess strategic leadership for implementation in order to advance understanding of leadership as a predictor of organizational context for implementation. The ILS also holds promise as a tool for leader and organizational development to improve EBP implementation.

## Introduction

The adoption, implementation, and sustainment of evidenced-based practices (EBPs) are becoming increasingly important for health and allied healthcare organizations and providers, and widespread adoption of EBPs holds promise to improve quality of care and patient outcomes [1,2]. Considerable resources are being allocated to increase the implementation of EBPs in community care settings with support for activities such as training service providers and increased staffing to support monitoring of implementation-related activities [3]. Although there are calls for increased attention to organizational context in EBP dissemination and implementation [4,5], there are gaps in examining how organizational context affects EBP implementation. Most relevant for this research is the need for development of measures to assess organizational constructs likely to impact implementation process and outcomes. One organizational factor in need of greater attention is that of leadership for EBP implementation [6].

Leaders can positively or negatively impact the capacity to foster change and innovation [7-10] and therefore are instrumental in facilitating a positive climate for innovation and positive attitudes toward EBP during implementation [6,11]. Although the role of leadership in EBP implementation is often discussed, it is rarely empirically examined. The limited empirical research in this area supports the presence of a relationship between general leadership ability and implementation of innovative practices [12], but focuses less on identifying specific behaviors that leaders may enact to facilitate EBP implementation. To stimulate and support additional empirical work in this area, there is a need for brief and efficient measures to assess specific actions leaders may engage in to influence the success of implementation efforts in their organizations or programs.

Both implementation and leadership theories emphasize the importance of leadership in supporting implementation of innovative practices such as EBP. For example, implementation scholars have asserted the importance of leadership in terms of obtaining funding, dispersing resources, and enforcing policies in support of implementation [13]. Research from the Collaboration for Leadership in Applied Health Research and Care has addressed the importance of leaders serving as clinical opinion leaders, managing implementation projects, fostering organizational learning climates, and obtaining senior management support [14]. Other research suggests that managers are responsible for interpreting research evidence, applying it to organizational contexts, and making research-informed implementation decisions [15]. Weiner’s organizational theory of innovation implementation suggests that leaders play a critical role in creating readiness for change, ensuring innovation-values fit, and developing plans, practices, structures, and strategies to support implementation [16].

There is also empirical evidence for the importance of leadership in predicting the success of implementation efforts. For example, transformational leadership (*i.e.*, the degree to which a leader can inspire and motivate others) has been shown to predict employees’ reported use of an innovative practice being implemented in their organization [12,17]. Consistent with transactional leadership (*e.g.*, providing contingent rewards) [18] perceived support from one’s supervisor has been associated with employees’ participation in implementation [19]. Much of the empirical research on leadership and implementation has focused on identifying mechanisms through which leaders affect implementation. These include a positive organizational climate [20], supportive team climate [21], and positive work attitudes [22]. Research has also focused on the role of leaders in influencing employee attitudes toward EBP [11] and commitment to organizational change [23].

Although general leadership is held to play an important role in implementation, research in this area has not necessarily outlined specific behaviors that leaders may enact in order to strategically influence followers to support the larger goal of successful implementation. Insight into such behaviors can be garnered from existing literature demonstrating that strategically-focused leadership predicts the achievement of specific goals. For example, a recent meta-analysis confirmed the relative advantage of strategic leadership—compared to general leadership—for specific organizational change initiatives [24]. Recent organizational research in climate for customer service [25] and climate for safety [26,27] has shown that strategically-focused leadership is a critical precursor to building a strategic climate, which subsequently predicts strategic outcomes such as increased customer satisfaction or decreased accidents, respectively.

Although more than 60 implementation strategies were identified in a recent review of the implementation literature [28], few focused on leadership as an implementation factor and none focused mainly on leader development to support EBP implementation. Of those identified, extant strategies involve the recruitment and training of leaders and involving leaders at different organizational levels [29-31]. Hence, we argue that leadership focused on a specific strategic imperative, such as adoption and use of EBP, can influence employee attitudes and behavior regarding the imperative. This is consistent with research demonstrating that leader and management support for implementation is a significant and strong predictor of positive implementation climate [32]. Thus, there is a need to identify those behaviors that leaders may enact to create a strategic EBP implementation climate in their teams and better facilitate the implementation and sustainment of EBP.

The goals of the present study were to develop a scale that focused on strategic leadership for EBP implementation and to examine its factor structure, reliability, and convergent and discriminant validity. We drew from strategic climate and leadership theory, implementation research and theory, implementation climate literature, and feedback from subject matter experts to develop items for the implementation leadership scale (ILS) to extend work on management support for implementation. In particular, we focused on leader behaviors related to organizational culture and climate embedding mechanisms that promote strategic climates [33]. In line with this literature, items were developed to assess the degree to which a leader is proactive with regard to EBP implementation, leader knowledge of EBP and implementation, leader support for EBP implementation, leader perseverance in the EBP implementation process, and leader attention to and role modeling effective EBP implementation. Through a process of exploratory factor analysis (EFA) followed by confirmatory factor analysis (CFA), we expected to find empirical support for the conceptual areas identified above. We also expected the final scale and subscales to demonstrate high internal consistency reliability. In regard to convergent validity, we expected that the derived leadership scale would have moderate to high correlations with other measures of leadership (*i.e.*, transformational and transactional leadership). Finally, in regard to discriminant validity, we expected to find low to moderate correlations between the derived leadership scale and a measure of general organizational climate.

## Method

### Item generation

Item generation and domain identification proceeded in three phases. First, as part of a study focused on developing an intervention to improve leadership for evidence-based practice implementation [18], the investigative team developed items based on review of literature relating leader behaviors to implementation and organizational climate and culture change [32,33]. Second, items were reviewed for relevance and content by subject matter experts, including a mental health program leader, an EBP trainer and Community Development Team consultant from the California Institute for Mental Health, and four mental health program managers. Third, potential items were reviewed by the investigative team and program managers for face validity and content validity. Twenty-nine items were developed that represented five potential content domains of implementation leadership: proactive EBP leadership, leader knowledge of EBP, leader support for EBP, perseverance in the face of EBP implementation challenges, and attention and role modeling related to EBP implementation.

### Participants

Participants were 459 mental health clinicians working in 93 different outpatient mental health programs in Southern California, USA. Of the 573 clinicians eligible to participate in this research, 459 participated (80.1% response rate). Participant mean age was 36.5 years (SD = 10.7; Range = 21 to 66) and the majority of respondents were female (79%). The racial/ethnic distribution of the sample was 54% Caucasian, 23.4% Hispanic, 6.7% African American, 5% Asian American, 0.5% American Indian, and 10% ‘other’. Participants had worked in the mental health services field for a mean of 8.5 years (SD = 7.7; Range = 1 week to 43 years), in child and/or adolescent mental health services for a mean of 7.5 years (SD = 7.6; Range = 1 week to 43 years), and in their present agency for 3.4 years (SD = 4.3; Range = 1 week to 28.1 years). Highest level of education consisted of 7% Ph.D./M.D. or equivalent, 68% master’s degree, 6.5% graduate work but no degree, 12.2% bachelor’s degree, 3% some college but no degree, and 0.7% no college. The primary discipline of the sample was 47% marriage and family therapy, 26% social work, 16% psychology, 3% child development, 2% human relations, 1% nursing, and 4.8% other (*e.g.*, drug/alcohol counseling, probation, psychiatry).

### Procedure

The study was approved by the appropriate Institutional Review Boards prior to clinician recruitment and informed consent was obtained prior to administering surveys. The research team first obtained permission from agency executive directors or their designees to recruit their clinicians for participation in the study. Clinicians were then contacted either via email or in-person for recruitment to the study. Data were collected using online surveys or in-person paper-and-pencil surveys.

For online surveys, each participant was e-mailed an invitation to participate including a unique username and password as well as a link to the web survey. Participants reviewed informed consent and after agreeing to participate were able to access the survey and proceed to the survey items. Once participants logged in to the online survey, they were able to answer questions and could pause and resume at any time. The online survey took approximately 30 to 40 minutes to complete and incentive vouchers ($15 USD) were sent by email after survey completion.

In-person data collection occurred for those teams in which in-person data collection was preferred or would be more efficient. Paper surveys were administered during meetings at each of the participating program locations. In most cases, the research team reserved one hour for data collection during a regular clinical work group or team meeting. Research staff obtained informed consent, handed out surveys to all eligible participants, checked the returned surveys for completeness, and then provided an incentive voucher to each participant. For participants not present at in-person meetings, paper surveys were provided and were returned to the research team in pre-paid envelopes.

Teams were identified in close collaboration with agency administrators. It was of utmost importance that team members shared a single direct supervisor to properly account for dependence in the data for variables pertaining to leadership. It was also important that participants completed the survey questions pertaining to leadership based on the proper supervisor as identified by the agency administrators. Participants completing the online survey selected their supervisor from a dropdown menu of supervisors within their agency in the beginning of the survey. The supervisor’s name was then automatically inserted into all questions regarding leadership in order to ensure clarity in the target of all leadership questions. The research team verified the identified leader with organization charts.

For in-person data collection, participants were given paper-and-pencil surveys with their supervisor’s name pre-printed on the front page of the survey and in sections pertaining to general leadership and implementation leadership. Participants were instructed to answer all leadership questions about the supervisor whose name was printed on their survey. In cases where a participant noted that they reported to a different supervisor, this was clarified and the survey was adjusted if deemed appropriate.

## Measures

### Implementation leadership scale (ILS)

Item development for the ILS is described above. All 29 ILS items were scored on a 0 (‘not at all’) to 4 (‘to a very great extent’) scale.

### Multifactor leadership questionnaire (MLQ)

The MLQ [34] is one of the most widely researched measures of leadership in organizations. The MLQ includes the assessment of transformational leadership, which has been found in numerous studies to be associated with organizational performance and success (including attitudes toward EBP), as well as transactional leadership [11]. The MLQ has good psychometric properties including internal consistency reliability and concurrent and predictive validity. All items were scored on a 0 (‘not at all’) to 4 (‘frequently, if not always’) scale. Transformational leadership was measured with four subscales: idealized influence (α = 0.87, 8 items), inspirational motivation (α = 0.91, 4 items), intellectual stimulation (α = 0.90, 4 items), and individualized consideration (α = 0.90, 4 items). The MLQ also includes one subscale identified as best representing transactional leadership: contingent reward (α = 0.87, 4 items).

### Organizational climate

The Organizational Climate Measure (OCM) [35] consists of 17 scales capturing the four domains of the competing values framework [36]: human relations, internal process, open systems, and rational goal. We utilized the autonomy (α = 0.67, 5 items) scale from the human relations domain, the formalization scale (α = 0.77, 5 items) from the internal process domain, and the efficiency (α = 0.80, 4 items) and performance feedback (α = 0.79, 5 items) scales of the rational goal domain [35] as measures for assessing discriminant validity of the ILS. All OCM items were scored on a 0 (‘definitely false) to 3 (‘definitely true’) scale.

### Statistical analyses

In order to determine whether the data represented a unit-level construct (in this case, clinical treatment work groups or teams), we examined intraclass correlations (ICCs) and the average correlation within group (a_*wg*_) for each item. Agreement indices are used to assess the appropriate level of aggregation for nested data. Higher levels of agreement suggest that the higher level of aggregation is supported. This is relevant for the current study as clinicians were working within clinical work groups or teams led by a single supervisor.

Work group/team-level data was randomized within organization to be utilized for either the EFA (n = 229; k = 46 teams) or CFA (n = 230; k = 47 teams). Exploratory factor analysis was used to derive and evaluate the factor structure of the scale using IBM SPSS. Principal axis factoring was selected for factor extraction because it allows for consideration of both systematic and random error [37] and Promax oblique rotation was utilized for factor rotation as we assumed that derived factors would be correlated. Item inclusion or exclusion was based on an iterative process in which items with relatively low primary loadings (*e.g.*, < 0.40) or high cross-loadings (*e.g.*, > .30) were removed [37]. The number of factors to be retained was determined based on parallel analysis, factor loadings, and interpretability of the factor structure as indicated in the rotated solution. Parallel analysis is among the better methods for determining the number of factors based on simulation studies [38]. Parallel analysis was based on estimation of 1,000 random data matrices with values that correspond to the 95^th^ percentile of the distribution of random data eigenvalues [39,40]. The random values were then compared with derived eigenvalues to determine the number of factors. Confirmatory factor analysis was conducted using Mplus [41] statistical software adjusting for the nested data structure using maximum likelihood estimation with robust standard errors (MLR), which appropriately adjusts standard errors and chi-square values. Missing data were handled through full information maximum likelihood (FIML) estimation. Model fit was assessed using several empirically supported indices: the comparative fit index (CFI), the Tucker-Lewis index (TLI), the root mean square error of approximation (RMSEA), and the standardized root mean square residual (SRMR). CFI and TLI values greater than 0.90, RMSEA values less than 0.10, and SRMR values less than 0.08 indicate acceptable model fit [41-44]. Type two error rates tend to be low when multiple fit indices are used in studies where sample sizes are large and non-normality is limited, as in the present study [45].

Reliability was assessed by examining Cronbach’s alpha internal consistency for each of the subscales and the total scale. Item analyses were also conducted, including an examination of inter-item correlations and alpha if item removed. Convergent and discriminant validity were assessed by computing Pearson Product Moment Correlations of ILS subscale and total scale scores with MLQ and OCM subscale scores.

## Results

Examination of distributions for all scale items indicated that data were generally normally distributed with no extreme skewness. Thus, we treated variables as continuous in our analyses. The presence of missing data was minimal. For example, among the 459 respondents, only 26 (6%) had any missing data. Of those with missing data, 17 of the 26 (65%) had missing information on only one item, two had two or three items missing (8%), and the remaining 7 (27%) had more than three items missing. For the EFA, we used bivariate (rather than listwise) deletion in order to minimize the number of excluded cases and used FIML estimation to address missing values in the CFA.

### Aggregation analyses

We first examined the amount of dependency among observations within groups using intraclass correlations (ICC, type 1) [46]. As shown in Table 1, the ICCs indicated a moderate degree of dependency among service provider responses within the same team. Nevertheless, the true variance tends to be underestimated whenever ICCs take on non-zero values, an effect that is magnified with increasing average cluster size [47]. However, the average cluster size was relatively small in this study (mean = 6), mitigating this concern.

**Table 1:** Implementation leadership scale, subscale and item statistics

								**EFA factor loadings**			
**ILS items, subscales, and total**	***Mean***	***sd***	***ICC***	***a***_***wg***_	***ev***	***v***	***α***	**1**	**2**	**3**	**4**
**1. Proactive leadership**	**2.12**	**1.25**	0.25	**0.68**	9.50	79.0%	0.95				
Established clear standards for implementation of EBP	2.16	1.33		0.67				**0.96**	0.02	0.06	−0.08
Developed a plan to facilitate EBP implementation	2.12	1.29		0.70				**0.95**	0.00	−0.05	0.05
Removed obstacles to implementation of EBP	2.09	1.30		0.67				**0.75**	0.02	0.07	0.12
**2. Knowledgeable leadership**	**2.56**	**1.18**	0.26	**0.72**	0.76	6.3%	0.96				
Knows what he/she is taking about when it comes to EBP	2.58	1.22		0.73				0.09	**0.94**	−0.03	−0.02
Is knowledgeable about EBP	2.59	1.20		0.71				−0.06	**0.87**	0.09	0.06
Is able to answer staff questions about EBP	2.50	1.25		0.71				0.30	**0.85**	0.06	0.04
**3. Supportive leadership**	**2.63**	**1.15**	0.22	**0.69**	0.49	4.1%	0.95				
Supports employee efforts to use EBP	2.63	1.21		0.70				0.02	0.10	**0.84**	0.02
Supports employee efforts to learn more about EBP	2.67	1.18		0.72				−0.03	−0.01	**0.83**	0.16
Recognizes and appreciates employee efforts	2.59	1.25		0.67				0.17	0.17	**0.69**	−0.08
**4. Perseverant leadership**	**2.36**	**1.25**	0.29	**0.69**	0.37	3.1%	0.96				
Perseveres through the ups and downs of implementing	2.37	1.29		0.69				0.05	0.07	0.05	**0.81**
Carries on through the challenges of implementing EBP	2.38	1.31		0.69				0.10	0.02	0.11	**0.78**
Reacts to critical issues regarding implementation of EBP	2.32	1.30		0.69				0.29	0.18	0.09	**0.44**
**Implementation leadership scale total**	2.42	1.12	0.29	0.70			0.98				

Note: *N* = 459 for means and standard deviations and ICC; *n* = 229 for other EFA derived statistics; *sd* = Standard deviation; ICC = intraclass correlation; *a*_*wg*_ = average within group correlation; *ev* = initial eigenvalue; *v* = variance accounted for before rotation; *α* = Cronbach’s alpha; bold font for means and sd indicate the overall scale mean and sd; bold font for EFA factor loadings indicates the scale on which the items load.

We next examined the average agreement within clinical work group for individual items and scales using a_*wg*(1)_ and a_*wg*(J)_, respectively [48-50]. a_*wg*_ ranges from 1 to −1, with a_*wg*(1)_ calculated as one minus the quotient of two times the observed variance divided by the maximum possible variance, and a_*wg*(J)_ is the sum of a_*wg*(1)_ values for items divided by the number of items for a scale. These statistics have the advantage over r_*wg*_[48,49] of not being scale and sample size dependent, and not assuming a uniform distribution [48,49]. Values of a_*wg*_ greater than 0.60 represent acceptable agreement and values of 0.80 and above represent strong agreement [48-50]. As shown in Table 1, considering ICCs and a_*wg*_, ILS items and scales should be considered as representing unit-level (*i.e.*, clinical work group or team) constructs in this study.

### Exploratory factor analysis

An iterative approach was taken to conducting the factor analyses and item reduction. In the first iteration and consistent with our hypotheses, five factors were specified and all 29 items were included. The EFA results showed that no items met the factor loading criteria for a proposed fifth factor (*i.e.*, no loadings > 0.40). That, coupled with the parallel analysis, suggested a four factor solution. Thus, we conducted the next EFA specifying four factors. The results suggested the removal of 15 items. Thirteen items were removed because of low primary factor loadings and/or high cross loadings, and two items were removed because of overlapping content with other items. Thus, 14 items were retained. The next EFA included 14 items and specified four factors. Based on those results, two additional items were removed due to statistical (*i.e.*, lower relative factor loadings) and conceptual (*i.e.*, item content less directly consistent with other items) criteria. The final EFA included 12 items with three items loading on each of four factors.

Table 1 displays the factor means, item means, ICC, a_wg_, initial eigenvalues, variance accounted for by each factor, internal consistency reliabilities, and rotated factor loadings. Internal consistencies were high, ranging from 0.95 to 0.98. Item analyses indicated that inter-item correlations were high, (range = 0.83 to 0.92) and the alpha for the subscales would not be improved by removing any items. As shown in Table 2, factor correlations ranged from 0.73 to 0.80, suggesting a higher order implementation leadership factor. The results of the CFA testing this higher order factor structure are provided in the next section. Subscale labels were created based on an examination of the items and factor loadings presented in Table 1. The first factor was labeled ‘Proactive Leadership’ as it indicated the degree to which the leader anticipates and addresses implementation challenges. Factor two addressed ‘Knowledgeable Leadership’ or the degree to which a leader has a deep understanding of EBP and implementation issues. Factor three was labeled ‘Supportive Leadership’ because it represented the leader’s support of clinicians’ adoption and use of EBP. Finally, factor four reflected ‘Perseverant Leadership’ or the degree to which the leader is consistent, unwavering, and responsive to EBP implementation challenges and issues.

**Table 2:** Implementation leadership scale factor intercorrelations

** Factor**	**1**	**2**	**3**	**4**
1. Proactive leadership	1.0			
2. Knowledgeable leadership	0.73	1.0		
3. Supportive leadership	0.75	0.79	1.0	
4. Perseverant leadership	0.79	0.77	0.80	1.0

Note: N = 229; All correlations, *p* < 0.001.

### Confirmatory factor analysis

**Figure 1:**
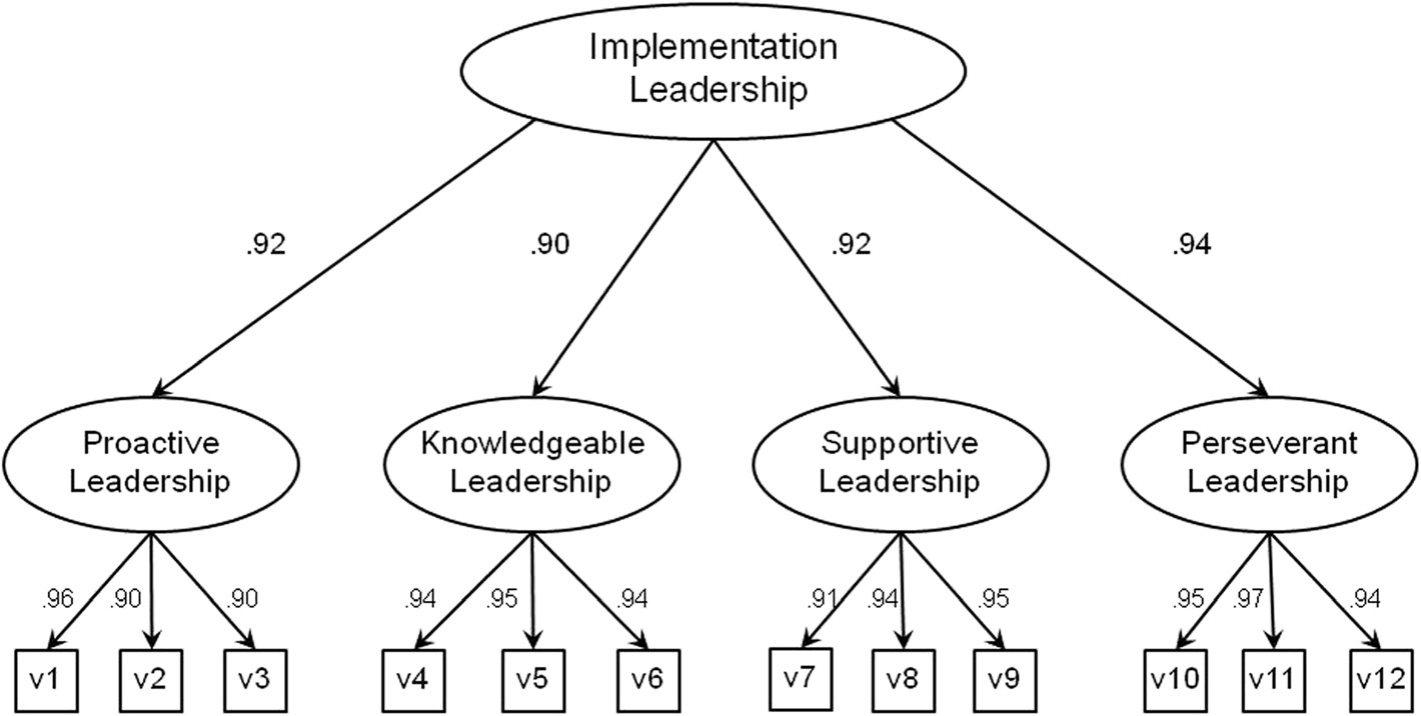
**Second-order confirmatory factor analysis factor loadings for the implementation leadership scale.** Note: n = 230; All factor loadings are standardized and are statistically significant, *p* < 0.001; *χ*^2^(50) = 117.255, *p* < 0.001; CFI = 0.973, TLI = 0.964; RMSEA = 0.076; SRMR = 0.034.

Confirmatory factor analysis was used with a sample independent of the EFA sample in order to evaluate the factor structure identified in the EFA above. In addition, because we proposed a higher-order factor model in which each subscale was considered an indicator of an overall implementation leadership latent construct, we evaluated the higher order model. We also controlled for the nested data structure (*i.e.*, clinicians within clinical work groups or teams). The higher order factor model demonstrated excellent fit as indicated by multiple fit indicators (n = 230; *χ*^2^(50) = 117.255, *p* < 0.001; CFI = 0.973, TLI = 0.964; RMSEA = 0.076; SRMR = 0.034). Figure 1 displays the standardized factor loadings for the higher-order factor model. First-order factor loadings ranged from 0.90 to 0.97, second-order factor loadings ranged from 0.90 to 0.94, and all factor loadings were statistically significant (*p*’s < 0.001).

### Convergent validity

Table 3 shows that, as predicted, the ILS scale scores had moderate to high correlations with MLQ subscales representing transformational and transactional leadership. Correlations ranged from 0.62 to 0.75 indicating convergent validity. The magnitude of the correlations suggests that leadership is being assessed by the ILS and that transformational leaders are likely to perform the behaviors necessary for effective EBP implementation, but not so high as to suggest that the MLQ and ILS scales are measuring identical constructs.

**Table 3:** Pearson product moment correlations of implementation leadership scale scores with multifactor leadership questionnaire [convergent validity] and organizational climate measure [discriminant validity] scores

	**Implementation leadership scales**				
	**Proactive**	**Knowledge**	**Support**	**Perseverant**	**ILS total**
MLQ					
Transformational leadership					
Intellectual stimulation	0.628**	0.698**	0.718**	0.699**	0.736**
Inspirational motivation	0.655**	0.683**	0.708**	0.705**	0.741**
Individualized consideration	0.618**	0.665**	0.705**	0.672**	0.715**
Idealized influence	0.658**	0.715**	0.721**	0.708**	0.753**
Transactional leadership					
Contingent reward	0.631**	0.644**	0.684**	0.649**	0.702**
Climate					
Autonomy	0.050	0.136*	0.121*	0.080	0.103*
Formalization	0.161**	0.147*	0.175**	0.156**	0.176**
Efficiency	0.227**	0.225**	0.281**	0.248**	0.268**
Feedback	0.327**	0.376**	0.406**	0.346**	0.392**

Note: N = 459; MLQ = Multifactor Leadership Questionnaire; Climate = General Organizational Climate Measure; *p < 0.05, **p < 0.01.

### Discriminant validity

Table 3 shows the results of the discriminant validity analyses. As predicted, the ILS scale scores had low correlations with OCM subscales representing aspects of general organizational climate. Correlations ranged from 0.050 to 0.406 indicating strong support for the discriminant validity of the ILS in contrast to general organizational climate.

## Discussion

The current study describes the development of the first measure of strategic leadership for evidence-based practice implementation, the ILS. We used an iterative process to develop items representing implementation leadership and then used quantitative data reduction techniques to develop a brief measure that may be easily and efficiently used for research and applied purposes. Such brief measures are needed to improve the efficiency of services and implementation research [51].

Although we originally proposed five factors of implementation leadership, quantitative analyses supported a four-factor model. The identified factors correspond to four of the original five subdomains originally conceived by the research team. The factors or subscales of the ILS represent Proactive Leadership, Supportive Leadership, Knowledgeable Leadership, and Perseverant Leadership. The factor that was not supported in these analyses had to do with the events and practices that leaders pay deliberate attention to as well as the extent to which a leader models effective EBP implementation. It may be that these behaviors are more akin to a strategic climate for EBP implementation and thus may have been less relevant for the core focus on leadership in the ILS. In addition, employees may not consciously recognize the specific targets of their leaders’ intentions. Conversely, it may be that the items that were developed did not sufficiently capture this aspect of leadership. Future studies should examine the degree to which leader attention and role modeling can be captured through the development of measures of organizational climate for EBP implementation.

The ILS demonstrated strong internal consistency reliability, convergent validity, and discriminant validity. Given that the ILS is very brief (*i.e.*, 12 items), administration and use in health services and implementation studies can be very efficient with little respondent burden. It generally takes less than five minutes to complete scales of this length. The practicality of this brief scale is consistent with calls for measures that can be utilized in real-world settings where the efficiency of the research process is paramount [52].

This is the first scale development study for implementation leadership and thus represents the first few phases (*i.e.*, qualitative item generation, exploratory factor analysis, confirmatory factor analysis, reliability assessment, validity assessment) of this line of research. However, the item and scale development was based on extant literature as well as investigator and practitioner knowledge and experience with leadership development and EBPs in community-based mental health service settings. Further research is needed to determine the utility of the measure for research and practice in this and other health and allied health care settings and contexts.

This study raises additional directions for future research. The factor analytic approach utilized here was highly rigorous. Not only did we randomize respondent data, but we randomly assigned data at the work group/team level to either the EFA or CFA analyses. Thus, there is no overlap in team membership across the two phases of this study. In addition, our examination of scale reliability and convergent and discriminant validity in this study confirmed expected relationships between the ILS and other constructs. For example, the moderate to high correlations with other leadership scales affirms that there is some overlap between implementation leadership and effective general leadership (*i.e.*, transformational and transactional leadership) but that unique aspects of leadership are also being captured. On the other hand, we had only one other measure of leadership in the study and future research should examine the degree to which other conceptual approaches and measures of leadership are associated with the ILS and its subscales [53]. In addition, the low association with general organizational climate suggests that the ILS dimensions are distinct from common measures of general organizational climate. Future research should examine the association of ILS scales with other measures of organizational climate and strategic climate for EBP implementation.

The ILS may help to inform our understanding of the influences and effects of leadership focused on EBP implementation. The ILS could also be utilized as a measure to identify leaders or to identify areas to develop in existing leaders. This is in keeping with an implementation strategy recently developed and pilot tested by the authors focused specifically on leadership and organizational change for implementation (LOCI) [18]. The LOCI implementation strategy utilizes data to support leader development and cross-level congruence of leadership and organizational strategies that support a first-level leader in creating a positive EBP implementation climate and implementation effectiveness [32,54]. Such strategies address calls for leadership and organizational change strategies to facilitate EBP implementation and sustainment [18].

The ILS is a brief tool that may be used in implementation research to assess the extent to which leaders support their staff in implementing EBP. The ILS and scoring instructions can be found in Additional files 1 and 2, or may be obtained from GAA. After establishing this baseline level of implementation leadership, researchers and/or organizations may apply this knowledge to the identification of areas for implementation leadership development. Because the scale is comprised of behaviorally focused items, results of the assessment may be used to guide leadership development. Thus, not only does this measure allow for assessment of implementation leadership, it has the potential to serve as a developmental tool to improve both leadership and EBP implementation success within organizations.

## Conclusions

The current study builds on previous research by extending the general concept of leadership to a new construct: strategic leadership for EBP implementation. This study suggests that effective leaders of EBP implementation should be proactive, knowledgeable, supportive, and perseverant in the implementation process. The extent to which these newly identified aspects of EBP leadership can impact individual factors (*e.g.*, employee behaviors), organizational factors (*e.g.*, implementation climate), and implementation outcomes should be the subject of future studies [54]. More immediately, strategies for improving leadership knowledge, skills, abilities, and behaviors in order to promote strategic climates that will improve the efficiency of EBP implementation should be developed and tested. In addition, the extent to which leadership influences fidelity and adoption of EBPs should be examined to increase our understanding of the complex ways in which leadership may affect clinician behavior in healthcare organizations. Pursuing such a research agenda has the potential to improve the efficiency and effectiveness of implementation efforts and to improve the reach and public health impact of evidence-based treatments and practices.

## Competing interests

GAA is an Associate Editor of *Implementation Science*; all decisions on this paper were made by another editor. The authors declare that they have no other competing interests.

## Authors’ contributions

GAA and MGE were study principal investigators and contributed to the theoretical background and conceptualization of the study, item development, study design, writing, data analysis, and editing. LRF contributed to the item development, study design, data collection, writing, and editing. All authors read and approved the final manuscript.

## Supplementary Material

Additional file 1 Implementation Leadership Scale (ILS). Click here for file

Additional file 2 Implementation Leadership Scale Scoring Instructions. Click here for file
