# Supplementary material for: The implementation leadership scale (ILS): development of a brief measure of unit level implementation leadership
Source: Implement Sci. 2014 Apr 14;9:45. doi: 10.1186/1748-5908-9-45 (PMC4022333; doi:10.1186/1748-5908-9-45)
Supplement: Additional file 1 — Implementation Leadership Scale (ILS). [file 1748-5908-9-45-S1.pdf]

Implementation Leadership Scale (ILS)

Gregory A. Aarons
gaarons@ucsd.edu

Mark Ehrhart
mehrhart@mail.sdsu.edu

Lauren Farahnak
lfarahnak@ucsd.edu

The ILS assesses the degree to which a leader is Proactive, Knowledgeable, Supportive, and Perseverant in regard to evidence-based practice implementation. There are two versions of the ILS, one for staff to report about their supervisor/leader, and another for supervisors/leaders to report about themselves.

Reference

Aarons, G.A., Ehrhart, M.G., & Farahnak, L.R. (2014). The Implementation Leadership Scale (ILS): Development of a Brief Measure of Unit Level Implementation Leadership. *Implementation Science.*

For information contact Gregory Aarons: gaarons@ucsd.edu

Staff Version

Please indicate the extent to which you agree with each statement.

0	1	2	3	4
**Not at all**	**Slight extent**	**Moderate extent**	**Great extent**	**Very great extent**

Proactive

1. [Name of Supervisor] has developed a plan to facilitate implementation of evidence-based practice 0 1 2 3 4

2. [Name of Supervisor] has removed obstacles to the implementation of evidence-based practice 0 1 2 3 4

3. [Name of Supervisor] has established clear department standards for the implementation of evidence-based practice 0 1 2 3 4

Knowledgeable

4. [Name of Supervisor] is knowledgeable about evidence-based practice 0 1 2 3 4

5. [Name of Supervisor] is able to answer my questions about evidence-based practice..... 0 1 2 3 4

6. [Name of Supervisor] knows what he or she is talking about when it comes to evidence-based practice 0 1 2 3 4

Supportive

7. [Name of Supervisor] recognizes and appreciates employee efforts toward successful implementation of evidence-based practice 0 1 2 3 4

8. [Name of Supervisor] supports employee efforts to learn more about evidence-based practice 0 1 2 3 4

9. [Name of Supervisor] supports employee efforts to use evidence-based practice 0 1 2 3 4

Perseverant

10. [Name of Supervisor] perseveres through the ups and downs of implementing evidence-based practice 0 1 2 3 4

11. [Name of Supervisor] carries on through the challenges of implementing evidence-based practice 0 1 2 3 4

12. [Name of Supervisor] reacts to critical issues regarding the implementation of evidence-based practice by openly and effectively addressing the problem(s) 0 1 2 3 4

Supervisor Version

Please indicate the extent to which you agree with each statement.

0	1	2	3	4
**Not at all**	**Slight extent**	**Moderate extent**	**Great extent**	**Very great extent**

Proactive

1. I have developed a plan to facilitate implementation of evidence-based practice...........	0 1 2 3 4
2. I have removed obstacles to the implementation of evidence-based practice	0 1 2 3 4
3. I have established clear department standards for the implementation of evidence-based practice	0 1 2 3 4

Knowledgeable

4. I am knowledgeable about evidence-based practice	0 1 2 3 4
5. I am able to answer staff's questions about evidence-based practice	0 1 2 3 4
6. I know what I am talking about when it comes to evidence-based practice	0 1 2 3 4

Supportive

7. I recognize and appreciate employee efforts toward successful implementation of evidence-based practice..	0 1 2 3 4
8. I support employee efforts to learn more about evidence-based practice	0 1 2 3 4
9. I support employee efforts to use evidence-based practice	0 1 2 3 4

Perseverant

10. I persevere through the ups and downs of implementing evidence-based practice	0 1 2 3 4
11. I carry on through the challenges of implementing evidence-based practice................	0 1 2 3 4
12. I react to critical issues regarding the implementation of evidence-based practice by openly and effectively addressing the problem(s)	0 1 2 3 4
