# Supplementary material for: The implementation leadership scale (ILS): development of a brief measure of unit level implementation leadership
Source: Implement Sci. 2014 Apr 14;9:45. doi: 10.1186/1748-5908-9-45 (PMC4022333; doi:10.1186/1748-5908-9-45)
Supplement: Additional file 2 — Implementation Leadership Scale Scoring Instructions. [file 1748-5908-9-45-S2.pdf]

Implementation Leadership Scale (ILS)

Abbreviated Items, Factor Loadings, Cronbach's Alphas, and Scoring

Gregory A. Aarons — gaarons@ucsd.edu
Mark Ehrhart — mehrhart@mail.sdsu.edu
Lauren Farahnak — lfarahnak@ucsd.edu

The ILS assesses the degree to which a leader is Proactive, Knowledgeable, Supportive, and Perseverant in implementing evidence-based practice.

Reference

Aarons, G.A., Ehrhart, M.G., & Farahnak, L.R. (2014). The Implementation Leadership Scale (ILS): Development of a Brief Measure of Unit Level Implementation Leadership. *Implementation Science.*

Item #	Scale	EFA Factor Loading	CFA Factor Loading	Alpha
	**Scale 1: Proactive**		.92†	.95
1	Developed a plan to facilitate EBP implementation	.95	.96	
2	Removed obstacles to implementation of EBP	.75	.90	
3	Established clear department standards for implementation	.96	.90	
	**Scale 2: Knowledgeable**		.90†	.96
4	Is knowledgeable about EBP	.87	.94	
5	Is able to answer staff questions about EBP	.85	.95	
6	Knows what he or she is talking about when it comes to EBP	.94	.94	
	**Scale 3: Supportive**		.92†	.95
7	Recognizes and appreciates employee efforts	.69	.91	
8	Supports employee efforts to learn more about EBP	.83	.94	
9	Supports employee efforts to use EBP	.84	.95	
	**Scale 4: Perseverant**		.94†	.96
10	Perseveres through the ups and downs of implementing EBP	.81	.95	
11	Carries on through the challenges of implementing EBP	.78	.97	
12	Reacts to critical issues regarding implementation of EBP	.44	.94	
	**ILS Total**			.98

Note: † indicates higher order CFA factor loading

SCORING THE SCALES

The score for each subscale is created by computing a mean score for each set of items that load on a given subscale. For example, items 1, 2, and 3 constitute Scale 1.

COMPUTING THE TOTAL SCORE

A mean of the scale scores may be computed to yield the mean score for the total ILS.

For questions or more information you may contact Dr. Aarons by email at: gaarons@ucsd.edu
